# Automation of Copper-Mediated ^18^F-Fluorination of Aryl Pinacol Boronates Using 4-Dimethylaminopyridinium Triflate

**DOI:** 10.3390/molecules29143342

**Published:** 2024-07-16

**Authors:** Mikhail A. Nadporojskii, Viktoriya V. Orlovskaya, Olga S. Fedorova, Dmitry S. Sysoev, Raisa N. Krasikova

**Affiliations:** 1 Granov Russian Research Center of Radiology and Surgical Technologies, 197758 St. Petersburg, Russia; nadporojskii@mail.ru (M.A.N.); dssysoev@mail.ru (D.S.S.); 2 N.P. Bechtereva Institute of the Human Brain, 197022 St. Petersburg, Russia; orlovskaya@ihb.spb.ru (V.V.O.); fedorova@ihb.spb.ru (O.S.F.)

**Keywords:** PET, [^18^F]fluoride, Cu mediated, pyridinium sulfonates, 6-L-[^18^F]FDOPA, automation

## Abstract

Currently, the copper-mediated radiofluorination of aryl pinacol boronates (arylBPin) using the commercially available, air-stable Cu(OTf)2Py4 catalyst is one of the most efficient synthesis approaches, greatly facilitating access to a range of radiotracers, including drug-like molecules with nonactivated aryl scaffolds. Further adjustment of this methodology, in particular, the [^18^F]fluoride recovery step for the routine preparation of radiotracers, has been the focus of recent research. In our recent study, an organic solution of 4-dimethylaminopyridinium trifluoromethanesulfonate (DMAPOTf) was found to be an efficient PTC for eluting radionuclides retained on the weak anion exchange cartridge, Oasis WAX 1cc, employing the inverse sorption–elution protocol. Notably, the following Cu-mediated radiofluorination of arylBPin precursors in the presence of the Cu(OTf)2(Py)4 catalyst can be performed with high efficiency in the same solvent, bypassing not only the conventional azeotropic drying procedure but any solvent replacement. In the current study, we aimed to translate this methodology, originally developed for remote-controlled operation with manual interventions, into the automated synthesis module on the TRACERlab automation platform. The adjustment of the reagent amounts and solvents allowed for high efficiency in the radiofluorination of a series of model arylBPin substrates on the TRACERlab FXFE Pro synthesis module, which was adapted for nucleophilic radiofluorinations. The practical applicability of the developed radiofluorination approach with DMAPOTf elution was demonstrated in the automated synthesis of 6-L-[^18^F]FDOPA. The radiotracer was obtained with an activity yield (AY; isolated, not decay-corrected) of 5.2 ± 0.5% (n = 3), with a synthesis time of ca. 70 min on the TRACERlab FX N Pro automation platform. The obtained AY was comparable with one reported by others (6 ± 1%) using the same boronate precursor, while a slightly higher AY of 6-L-[^18^F]FDOPA (14.5 ± 0.5%) was achieved in our previous work using commercially available Bu_4_NOTf as the PTC.

## 1. Introduction

Positron emission tomography (PET) is a leading molecular imaging modality in clinical, preclinical, and biomedical research settings [1]. The wide and growing application of PET in combination with computed tomography (CT) in clinical oncology, cardiology, and neurology [2,3,4], as well as in drug development processes [5], has led to an increasing interest in new methods in radiochemistry. Among the conventional PET radionuclides, fluorine-18 (T_1/2_ 109.8 min, 97% β^+^) has been considered the most in demand due to its outstanding decay properties, such as low positron energy (0.635 MeV) and short range of positrons in tissue (2.4 mm), providing the highest theoretical spatial resolution of PET images. Radionuclides in the form of no-carrier-added aqueous [^18^F]fluoride can be reliably and routinely produced with high activity (up to 900 GBq) and high molar activity (A_m_) via an ^18^O(p,n)^18^F nuclear reaction in an ^18^O-water cyclotron target. The convenient cf. 2 h half-life of ^18^F allows for the multi-step synthesis and commercial distribution of radiopharmaceuticals to remote PET imaging sites that lack cyclotron and radiochemistry/radiopharmacy facilities. After irradiation, aqueous [^18^F]fluoride is trapped on a strong anion-exchange resin, such as quaternary methylammonium (QMA) cartridges, and then eluted, typically with an acetonitrile/water solution containing a complex of inorganic base and cryptand (K_2_CO_3_/kryptofix-K2.2.2. or tetrabutyl ammonium salts), and it is then dried via repeated azeotropic distillation with MeCN. The obtained highly nucleophilic [^18^F]fluoride is further involved in S_N_2 or S_N_Ar radiofluorination processes with the appropriate labeling substrates [6,7,8]. The vast majority of clinically relevant radiotracers are prepared via aliphatic nucleophilic S_N_2 fluorination methods, which are easily implemented into different types of automated apparatuses, including cassette-based synthesizers [9]. Over the past decade, substantial advances have been made in the development of so-called “late-stage” radiofluorination techniques [10,11,12,13], opening new possibilities for the direct introduction of the ^18^F-label into aromatic compounds with nonactivated aryl scaffolds. Among them, the copper-mediated ^18^F-fluorination of pinacol esters of aryl boronic acids (arylBPin), introduced by Gouverneur’s group [14] following the chemistry developed by Sanford’s group [15], is one of the most efficient synthetic approaches for the preparation of a range of radiotracers using commercially available, air-stable copper catalysts (for recent reviews, see [16,17,18]). The scope of the Cu-mediated fluorination reaction was further extended to boronic acids [19] and organostannanes [20].

However, the implementation of Cu-mediated radiofluorination for full-batch tracer production exposed the problem of the sensitivity of the process to basic conditions. As a consequence, substantial efforts were expended on designing novel protocols for reprocessing [^18^F]fluoride by introducing less basic or neutral phase-transfer catalysts (PTCs) in organic solvents [21] and the so-called “back-flushing protocol” [22], where ^18^F^−^ is loaded onto the ion-exchange cartridge from the male side instead of the female side and eluted in the opposite direction. Zlatopolskiy et al. [22] introduced the combination of this protocol with [^18^F]fluoride elution using a solution of tetraethylammonium bicarbonate (Et_4_NHCO_3_) in alcohol, as suggested by the same group [23], providing high recovery rates of the radionuclide from the ion-exchange cartridge and good fluorination efficiency of boronic ester precursors. Moreover, applying an alcoholic solution of the PTC in the [^18^F]F^−^ elution step and the same alcohol as a co-solvent in the following radiofluorination allowed for the operationally simple and practical azeotropic drying-free approach, facilitating automation. In current practice, this procedure, known as “alcohol-enhanced” Cu-mediated radiofluorination in DMA/*n*-BuOH mixture [23], has found broad application using various substrates [24,25,26,27,28,29].

In addition to the commercially available Et_4_NHCO_3_, Et_4_NOTf, Bu_4_NOTf, and other alkylammonium salts applied in Cu-mediated radiofluorinations, our group suggested 4-dimethylaminopyridinium trifluoromethanesulfonate (DMAPOTf) as an efficient eluting agent and PTC [30]. Preliminary studies revealed that a solution of DMAPOTf in organic solvent provided over 80% recovery of [^18^F]fluoride from weak anion-exchange resin (Oasis WAX 1 cc, 30 mg) when the “back-flushing” protocol was employed. Notably, the following Cu-mediated radiofluorination of arylBPin precursors using the Cu(OTf)_2_(Py)_4_ catalyst can be performed directly in the same solvent, bypassing not only the azeotropic drying procedure but any solvent replacement. This straightforward ^18^F-processing procedure was evaluated by fluorinating a range of arylBPin substrates using a remote-controlled apparatus (in-house design), and high RCC values ranging from 80 to 95% were obtained by carrying out radiofluorinations using DMA as the reaction solvent [30].

However, the attempts to employ DMAPOTf in the radio-labeling of complex arylBPin substrates were not so successful. For instance, Wenzel et al. [31] reported high RCCs (54–88%) obtained for the Cu-catalyzed labeling reaction of model [^18^F]4-fluorobiphenyl in DMA and DMI, while less than 1% RCC was found for the target product, ^18^F-labeled triazolopyridopyrazine-based derivative (PDE2A inhibitor), using the corresponding arylBPIn precursor. In addition to boronates, DMPAOTf has been recently tested in the radiofluorination of the stannyl precursor for the preparation of [^18^F]AG-120 in the presence of Cu(OTf)_2_(Py)_4_ [32]. In that study, [^18^F]fluoride retained on the Chromafix PS HCO_3_ cartridge was eluted using a solution of DMPAOTf in methanol; the subsequent radiofluorination in DMI resulted in an RCC in a range of 0.3–0.5%. At the same time, the RCC of 10% was obtained using the commercially available TBAOTf as a PTC, providing this novel radiotracer in the amounts sufficient for preclinical studies in a glioma model [31]. Alternatively, Zhang et al. [33] used DMAPOTf in combination with Cu(OTf)_2_ as the copper source. The RCC values obtained for various model substrates in this case varied from 23 to 87%; [^18^F]FMZ, the GABA_A_ receptors’ radioligand, was obtained on a preparative scale in 47% isolated RCY, with an average synthesis time of 60 min. However, to achieve high elution efficiency for [^18^F]fluoride retained on the strong anion-exchange cartridge Chromafix PS HCO_3_ (45 mg), the amount of the packing resin has to be reduced to a half. While this solution seems to be helpful for the research application, it hardly complies with GMP regulations in the routine production of radiotracers.

Based on all the above considerations, we opted for the use of the weak anion-exchange cartridge Oasis WAX 1 cc, in accordance with the previously developed protocol for Cu-mediated radiofluorination with DMAPOTf elution [30], with the aim to enhance the utility of this approach towards automated operation using the well-known TRACERlab automation platform (GE Health Care). The optimal elution and radiofluorination conditions were established by carrying out experiments on a series of model ArylBPin substrates; the practicality of the method was evaluated in the automated synthesis of 6-L-[^18^F]FDOPA, a highly demanding PET radiotracer with multiple clinical applications [34,35,36,37].

## 2. Results and Discussion

### 2.1. Radiofluorination of Model ArylBPin Substrates Using DMAPOTf as the PTC

The advancements made in the preparation of reactive [^18^F]fuoride for the nucleophilic synthesis of various PET radiotracers, including base-sensitive radiolabeling substrates, have been the subject of several recent reviews [21,38,39,40]. During the last decade, a number of innovative methods and techniques were introduced by different groups through optimization of the base and phase-transfer agent used, [^18^F]F- eluting solvent composition, conditioning of the anion-exchange cartridge prior to use, and other parameters [41]. Among them, the type of anion-exchange cartridge and the amount of packing material have proved to be important and substantially influence the efficacy of [^18^F]F^−^ elution, as well as the subsequent radiolabeling. Practically, the capture-and-release process is usually accomplished by using the standard Accell^TM^ Plus QMA light Sep-Pak cartridge (filled with 130 mg of quaternary ammonium chloride polymer) or the less common Chromafix^®^ PS-HCO_3_ (Sep-Pak cartridge filled with 46 mg of quaternary ammonium bicarbonate polymer). The barrel-type cartridges, such as Vac QMA 1 cc (100 mg), Oasis WAX 1 cc (30 mg), and others, are employed mostly for radiopharmaceutical research. In our previous study [30], Oasis WAX, a weak anion-exchange resin, was used in combination with 4-dimethylaminopyridinium trifluoromethanesulfonate (DMAPOTf) as an efficient PTC for the radiofluorinations of arylBPIn substrates catalyzed by Cu(OTf)_2_(Py)_4_. Considering this pyridinium sulfonate salt as a weak base, the WAX cartridge packed with the lowest amount of the absorbent material (WAX 1cc, 30 mg) was selected to facilitate the elution process. As a result, high recovery (EE c.a. 78%) was obtained using a solution of DMAPOTf in DMA that was one of the most suitable solvents for Cu-mediated radiofluorinations of arylBPin substrates [16,17,18]. Carrying out the elution process and fluorination reaction in the same solvent allows for the elimination of conventional azeotropic drying and solvent exchange steps, thus simplifying the synthesis work-up.

Alternatively, the strong anion-exchange cartridge Chromafix PS-HCO_3_ (45 mg) was tested with various eluting solvents [30]. A high recovery yield (EE of 84%) was obtained only for a methanolic solution of DMAPOTf; however, in MeOH, no ^18^F-radiofluorination occurred. Zhang et al. [33] reported the radiofluorination of arylBPIn precursors using DMAPOTf and Cu(OTf)_2_ as a copper source and [^18^F]F-processing on the Chromafix PS-HCO_3_ Sep-Pak cartridge. To achieve high elution efficiency with DMA or DMF solutions of the PTC (>95%), the packing material of the cartridge was reduced to half (~20 mg). However, this research solution does not comply with the GMP regulations on the routine production of radiotracers. Therefore, Oasis WAX 1 cc was found to be the best choice for Cu-catalyzed radiofluorination with DMAPOTf elution.

**Figure 1:**
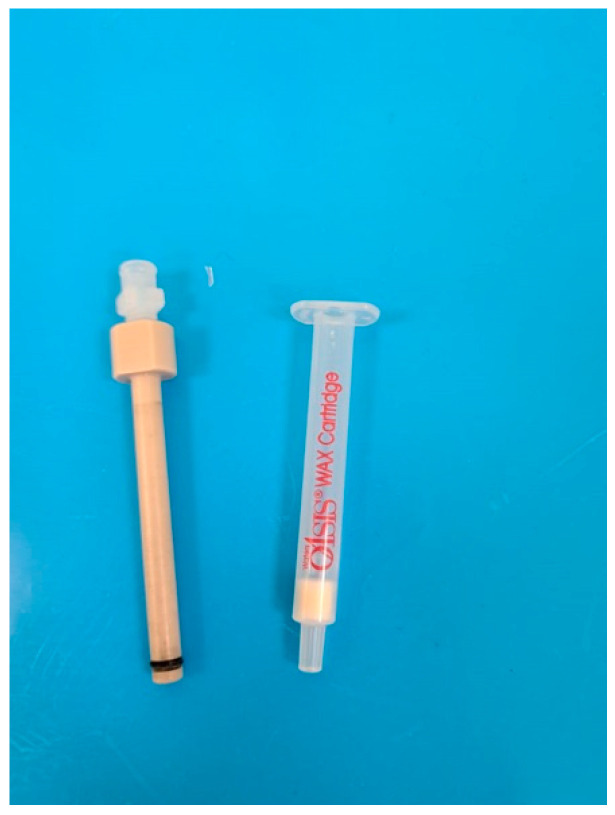
WAX 1cc cartridge (**right**) and PEEK liner with the Luer adapter (**left**).

Under remote-controlled operation, in small-scale radiolabeling experiments using DMAPOTF elution from OASIS WAX 1cc in DMA, RCCs in the range of 80–95% have been achieved for a wide range of arylBPin substrates [30]. The notable feature of the work-up procedure [29] is the use of a reverse-loading/elution protocol for ^18^F-preprocessing. This protocol starts with loading radionuclide onto the cartridge from the male side, followed by rinsing the resin with the solvent to remove residual water, and then elution of [^18^F]fluoride from the female side using a solution of the PTC in an appropriate solvent. This so-called “back-flushing protocol” suggested by Zlatopolskiy et al. [22] is quite compatible with the Sep-Pack-type cartridges implemented onto automated synthesis modules but not with the barrel-type cartridges such as WAX 1cc. The problem concerns the complete removal of water drops from the inner surface of the barrel from the male side. In contrast to manual operation, the water removal is associated with difficulties when a WAX 1 cc cartridge is integrated into the automation apparatus. To address this issue, we designed a PEEK liner (insert) that adheres closely to the walls of the barrel. The liner is equipped with a Luer-type female adapter at the top and a replaceable ring at the bottom (as shown in Figure 1). With this modification of the cartridge design, the residual water and/or rinsing solvent can be removed completely by carefully applying gas flow from the male side according to the “back-flushing protocol”.

**Figure 2:**
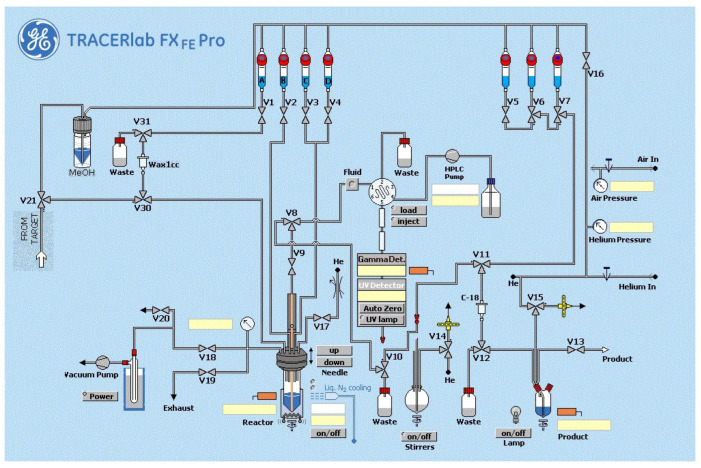
A schematic diagram of the GE TRACERlab FX_FE_-Pro module modified for nucleophilic fluorinations using aqueous [^18^F]fluoride. A: 20 μmol DMAPOTf in DMI/DMA 1/1 (0.6 mL); B: 80% EtOH (2 mL); C,D: empty vessels; reactor (RV)—20 μmol of precursor **1**–**7** and 20 μmol Cu(OTf)_2_(Py)_4_ in DMI (0.6 mL).

For the purpose of this study, we modified the TRACERlab FX_FE_ Pro synthesis module (GE Healthcare) originally designed for the electrophilic ^18^F-fluorinations using [^18^F]F_2_ gas, which is underutilized in our lab. Accordingly, the modified module configuration with additional components (valves, Luer adapters, etc.) was able to implement the [^18^F]fluoride “back-flushing” protocol. As shown in Figure 2, in the new configuration, three additional valves (V21, V30, V31) are added together with a V-shaped 5 mL Wheaton vial for the rinsing solvents. The input of the irradiated water-^18^O is connected to V21, while the modified WAX 1cc cartridge is placed into the line tubing between V30 and V31. The process sequence is described in Section 3.3 in the Experimental Section.

**Scheme 1:**
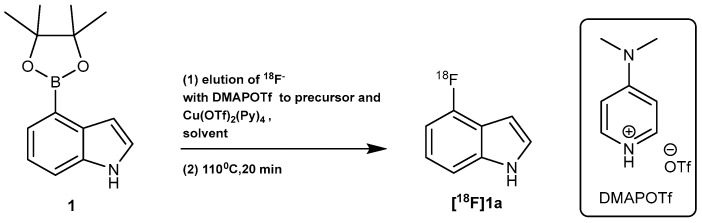
Copper-mediated radiofluorination of **1** using DMAPOTf as the PTC.

A set of different experimental conditions (elution solvent, reaction time and temperature, reaction solvent, amounts of Cu catalyst and precursor) were screened with the aim of identifying the key parameters that influence the RCC of the radiofluorination with DMAPOTf elution in the automated mode. Indole-4-boronic acid pinacol ester (**1**, Scheme 1) was selected as a model ArylBPin substrate.

Firstly, we tested the elution efficiency of [^18^F]fluoride trapped on the WAX 1cc cartridge with a solution of DMAPOTf in DMA. Our previous investigations highlighted that rinsing the cartridge with organic solvent to remove residual water prior to ^18^F-elution can significantly impact the radiofluorination efficiency [30]. In brief, the [^18^F]fluoride-trapped cartridge was rinsed with anhydrous solvent (1.5 mL), dried with nitrogen gas in the same direction, and then slowly eluted in the direction opposite to loading with a DMA solution of DMAPOTf into the reaction vessel prefilled with **1** and Cu mediator in DMA for direct radiofluorination. This straightforward protocol allows for the elimination of conventional azeotropic drying and solvent exchange steps, which simplifies the synthesis procedure. Of the several rinsing solvents investigated, methanol was found to be the best, providing high RCC and elution efficiency (Entry 5, Table 1). The low viscosity of MeOH provides additional benefits, facilitating the automated performance of the elution process.

As in the first published method [14] for the Cu-mediated fluorination of pinacol boronic esters, in most of the following studies, the reaction was carried out at 110 °C for 20 min, with access to the atmospheric oxygen typically required for this type of reaction [14]. Similar conditions were utilized in our previous work with DMAPOTf elution [30] and were reevaluated within this study. Four temperatures were screened (100, 110, 120, and 140 °C); the highest RCC was shown at 110 °C at a 20 min reaction time. The resulting temperature–RCC and time–RCC curves are displayed in the Supplementary Materials (Figures S1 and S2). Notably, in this study, the reaction mixture was not pressurized by air; however, access to the atmospheric oxygen was provided by reactants filling into the reaction vessel.

We next tested the effect of the precursor and Cu catalyst ratio and the amounts in the RCC in the model radiofluorination of **1** in DMA. Different precursor/catalyst amounts were investigated, with the precursor and Cu catalyst amounts increasing from 5 to 20 μmol and 5 to 30 μmol, respectively (Table 2). A precursor/catalyst ratio of 1:1 resulted in the highest RCCs of 75% when 20 μmol of each reactant was used (Entry 5, Table 2).

In addition to DMA, which was used in the previous study both as an eluting and reaction solvent [30], several other solvents were screened with the aim of enhancing the radiofluorination efficiency of **1**; fluorination was carried out with the established optimal amounts of precursor and Cu mediator (Table 3).

**Figure 3:**
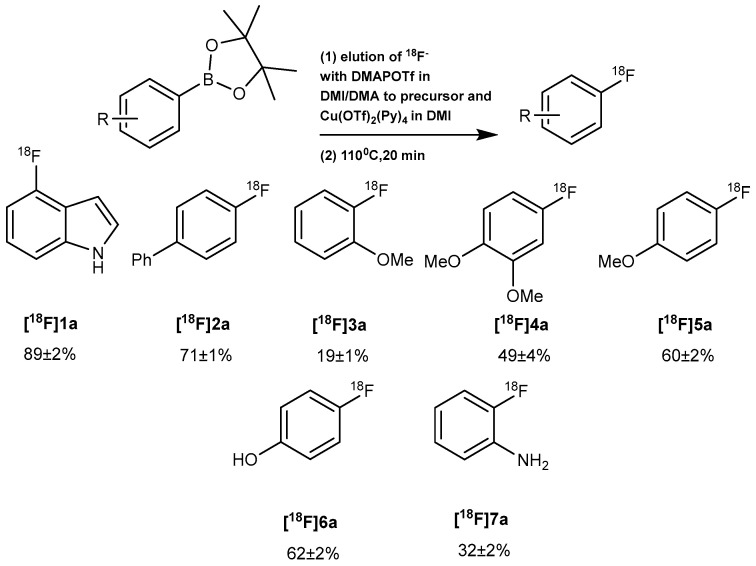
Substrate scope study; reaction conditions: 25 µmol of DMAPOTf, 20 µmol of arylBPin substrate, 20 µmol of Cu(OTf)_2_(Py)_4_, 1.2 mL of DMA/DMI 1/4, 110 °C, 20 min.

The highest RCC (92%) was achieved carrying out radiofluorination in the neat DMI; however, this solvent was not as efficient on the elution process, providing only 58% EE (Entry 5, Table 3). A decent balance between EE and RCC was observed using DMA as an eluting solvent and DMI in the radiofluorination step (Entry 7, Table 3). Further evaluation revealed that the reaction performance could be improved by replacing the elution solvent with a mixture of equal amounts of DMA and DMI (1/1); in that case, the fluorination reaction proceeds under an excess of DMI (DMA/DMI 1/4) (Entry 8, Table 3). Under these conditions, the highest RCC (c.a. 90% (Supplementary Materials, Figure S1)) was achieved while maintaining high elution efficiency. Finally, this optimized protocol was tested in a substrate scope study of various pinacol boronic esters (Figure 3).

Most of the screened aromatic substrates underwent radiofluorination under our conditions in moderate to high RCCs. However, the radiofluorination efficiencies were somewhat lower compared with our original report on small-scale reactions with DMAPOTf elution under remote-controlled operation [30]. This discrepancy in the results can be attributed to the different reaction solvents and reagent amounts used and to the design and volumes of the reaction vessels: a 15 mL volume round-bottom reactor in a TRACERLab FX_FE_ Pro vs. the 5 mL V-shaped reaction vessel (Wheaton vial) used in the remote-controlled apparatus [30].

### 2.2. Radiosynthesis of 6-L-[^18^F]FDOPA Using DMAPOTf as the PTC

#### 2.2.1. Radiolabeling Approach

**Scheme 2:**
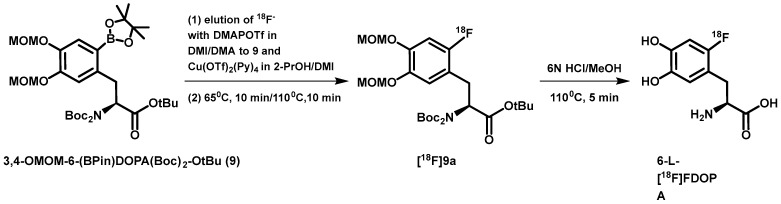
Radiosynthesis scheme for 6-L-[^18^F]FDOPA via Cu-mediated radiofluorination of **8**.

Following our preliminary results on the automated radiofluorination procedure for model arylBPin substrates, the methodology was extended to demonstrate its usefulness in the automated production of 6-L-[^18^F]FDOPA, a highly demanding PET radiotracer for the diagnosis of Parkinson’s disease and neuroendocrine and cerebral tumors [34,35,36,37]. We selected radiofluorination of the 3,4-OMOM-6-(BPin)DOPA(Boc)_2_-OtBu labeling precursor (**8**, Scheme 2) in the presence of Cu(OTf)_2_(Py)_4_, followed by acid hydrolysis from several radiolabeling protocols that have been suggested for Cu-mediated preparation of 6-L-[^18^F]FDOPA, employing ArylBPin precursors [16,24,42,43]. This two-step synthesis route for 6-l-[^18^F]FDOPA was applied in our previous study [24] using an alcohol solution of tetrabutylammonium triflate (Bu_4_NOTf) as a neutral PTC. Employing the “back-flushing” protocol leads to both high recovery rates of ^18^F-fluoride from the Sep-Pak Accell Plus QMA Plus Light cartridge and high RCC (>80%) in the subsequent radiofluorination of **8** (90 °C, 20 min in 2-PrOH/acetone under nitrogen atmosphere). The 6-l-[^18^F]FDOPA was isolated from the reaction mixture with an AY of 14.5 ± 0.5% (AY, isolated activity yield, not corrected for radioactive decay). With the same labeling precursor **8** and with Cu(OTf)_2_ as a copper source (for in situ formation of the corresponding Cu complex with pyridine), Scott’s group [43] performed GMP-compliant synthesis of 6-L-[^18^F]FDOPA on a GE TRACERlab FX_FN_ automated platform furnishing a radiotracer in the AY of 6 ± 1%.

Motivated by our results on the radiofluorination of model arylBPin substrates in the automated mode, we intend to test DMAPOTf in conjunction with WAX 1cc as a weak anion-exchange cartridge for its efficiency in the automated synthesis of 6-L-[^18^F]FDOPA via Cu-mediated fluorination of **8**.

#### 2.2.2. Optimization of the Radiofluorination Step

The initial selection of radiolabeling conditions for **8** was based on the results obtained in the radiofluorination of **1** presented in Table 3. Model experiments with this arylBPin substrate revealed that the solution of DMAPOTf in DMI/DMA (1/1) was the best eluting agent for the recovery of [^18^F]F^−^ retained on the Oasis WAX 1 cc-modified cartridge (Figure 1). Rinsing the cartridge prior to the release of the radionuclide with MeOH was an integral part of the “back-flushing” ^18^F-processing protocol applied. In the optimization studies, two automated platforms were employed: the commercially available TRACERlab FX_FE_ Pro synthesizer (FX FE, Table 4) adapted for nucleophilic fluorinations (Figure 2) and a remote-controlled apparatus (in-house development). Under remote-controlled operation, the reactions were carried out in 5 mL V-shaped vials with a screw cap (Wheaton) placed into a heating block, under magnetic stirring, while the transfer of all the reagents was achieved through nitrogen flow. The module design is described in detail elsewhere [44,45].

Initial experiments on the radiofluorination of **8** under the optimal conditions established for model arylBPin substrates (DMI/DMA 4/1, 110 °C, 20 min, no air atmosphere), however, resulted in poor RCC (Entry 1, Table 4). The increased precursor amount did not provide a substantial improvement (Entry 2, Table 4); however, changing the reaction solvent for DMI/2-PrOH has been found to be effective (Entries 3,4, Table 4). The beneficial effect of alcohols as co-solvents in Cu-mediated radiofluorinations of arylBPin substrates, first discovered by Zischler and coworkers [23], has been found to be useful in the preparation of a wide range of radiotracers, including ring-fluorinated aromatic amino acids [23,24,25,26,27]. As a refinement of the method, we implemented a two-step heating protocol: the reaction mixture was heated at 65 °C for 10 min, followed by a second round of heating at 110 °C for 10 min, while the reactor was sealed (Entries 5–7, Table 4). This methodology was recently introduced by our group in the Cu-mediated radiofluorination of a boronic acid precursor, providing a high radiochemical yield of 6-[^18^F]fluoropiperonal, the prosthetic group in the synthesis of [^18^F]anle138b [45]. Implementation of this two-step heating procedure provided the highest RCC, of [^18^F]**8a**, in the range of 65–77% (Entries 5–7).

#### 2.2.3. Automated Synthesis of 6-L-[^18^F]FDOPA

As shown above, the first synthesis step—the radiofluorination of **8** dissolved in DMI/2-PrOH—furnished protected 6-L-[^18^F]FDOPA (**8a**) in ca. 70% RCC (Supplementary Materials, Figure S2). The subsequent deprotection step (Scheme 1) has always been a challenge in this synthesis and is associated with difficulties in automation. Cleavage of the catechol-protective groups commonly employed in the synthesis of 6-L-[^18^F]FDOPA, such as the 4,5-methylenedioxy or 4,5-*bis*-methoxy groups, requires harsh reaction conditions (57% aq. HI, 180–200 °C, 20 min) [15], which are not compatible with most automated synthesis modules. Gratifyingly, due to easy-to-cleave MOM protecting groups on the catechol moiety and a tert-butyl ester group protecting the amino acid fragment in the structure of **8**, the hydrolysis/deprotection step can be performed using less-aggressive aqueous HCl solutions. In our previous work on the synthesis of 6-L-[^18^F]FDOPA [24], deprotection was performed via treatment of the radiofluorinated intermediate **8a** with 6N HCl/MeOH at 110 °C for 5 min in the presence of ascorbic acid. However, we observed incomplete hydrolysis of the intermediary radiolabeled **8a** when the acid was directly added to the radiofluorination reaction mixture (2-PrOH/acetone); therefore, the solvents had to be evaporated [24]. In the current study, the removal of high-boiling-point fluorination solvents (DMA, DMI) prior to hydrolysis would require substantial effort and time. To eliminate the solvents, the diluted reaction mixture was passed through the Sep-Pak C18 cartridge, followed by the release of **8a** in 1.5 mL MeOH. The eluate was mixed with 6 N HCl (0.4 mL) and 0.25 M ascorbic acid (0.2 mL), and the hydrolysis was carried out for 5 min at 110 °C under stirring in a nitrogen flow with an open waste and then for 5 min with a closed waste. Completeness of the hydrolysis/deprotection step was confirmed via radioHPLC analysis of the reaction mixture (HPLC system 1).

The reaction conditions described above for the radiofluorination and hydrolysis/deprotection steps were established while running the synthesis on the remote-controlled apparatus. To develop a fully automated procedure, these conditions were translated to the commercially available automated synthesis module TRACERlab FX N Pro (GE Healthcare) that was equipped with two reaction vessels needed for the two-step radiosynthesis with intermediate purification. In brief, after the trapping and elution of [^18^F]fluoride (10–11 GBq) from WAX 1cc using a solution of DMAPOTf (25 μmol in 0.6 mL of DMI/DMA 1/1), the labeling reaction of [^18^F]DMAPF with precursor **8** (20 μmol) was performed in DMA/DMI/2-PrOH (total volume 1.2 mL) via two-step heating (Scheme 1). Notably, radiofluorination proceeds under a nitrogen atmosphere instead of the air atmosphere usually applied for the Cu-mediated ^18^F-fluorination of boronates [14]. RadioTLC analysis of an aliquot of the crude reaction mixture (Supplementary Materials, Figure S3) revealed an almost 50% drop in the efficiency of radiofluorination for the same amounts of the reactants (an RCC of 35–45% vs. 70% in the remote-controlled module). This may be a consequence of the reaction vessel design of the TRACERlab FX N Pro automated module, which has a high volume/surface vs. the small volume of the reaction mixture. After dilution of the reaction mixture with 9 mL of water, the resulting mixture was directed onto the Sep-Pak C18 cartridge to eliminate the non-reacted [^18^F]fluoride and reaction solvents. Protected 6-L-[^18^F]FDOPA was recovered from the C18 cartridge in 1.5 mL of MeOH in the second reaction vessel. Then, the solvent was partly evaporated under a flow of N_2_ (110 °C, 3 min). The hydrolysis step was kept as described above: heating with 6 N HCl (0.4 mL) and ascorbic acid (0.25 M, 0.2 mL) at 110 °C for 3 min under stirring, and the volume of the resulting solution was reduced to 0.5 mL via evaporation under a flow of N_2_ at the same temperature. The resulting solution containing a crude product was diluted with 1.5 mL 0.1% CH_3_COOH to be injected into a 2 mL HPLC loop.

#### 2.2.4. Purification and Quality Control of 6-L-[^18^F]FDOPA

**Figure 4:**
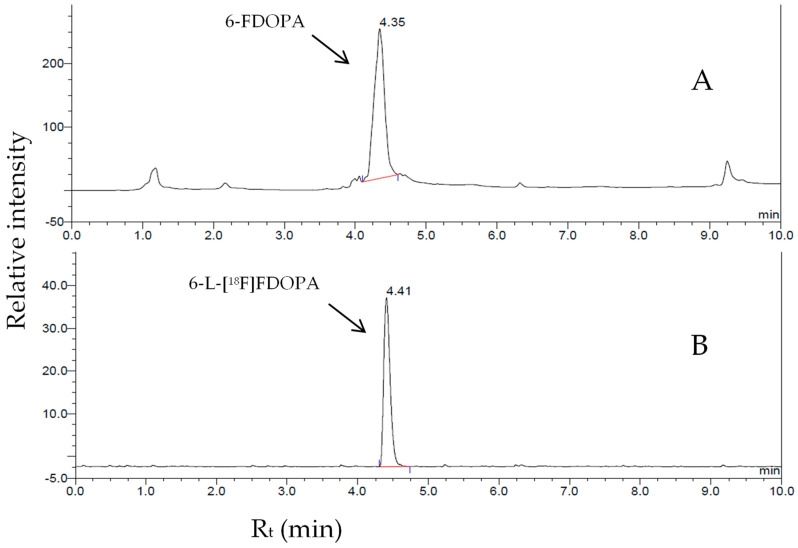
Representative HPLC chromatogram of formulated 6-L-[^18^F]FDOPA (gradient conditions, HPLC system 1, UV 254 nm). (**A**) UV trace of an authentic reference (254 nm); (**B**) radioactivity channel.

6-L-[^18^F]FDOPA was isolated from the reaction mixture via semipreparative reverse-phase HPLC using aqueous 0.1% CH_3_COOH (pH 4) as the mobile phase at a 4 mL/min flow rate on the Ascentis RP-AMIDE (5 μm, 250 × 10 mm) column. The product fraction (R_t_ 12–13 min, 4–5 mL volume, Supplementary Materials, Figure S4) corresponding to pure 6-L-[^18^F]FDOPA was collected in a vented sterile vial pre-filled with 5 mL of normal saline, following on-line sterilization through the 0.22 μm filter. 6-L-[^18^F]FDOPA was obtained with a radiochemical purity above 99% (confirmed via both radioHPLC (Figure 4) and radioTLC (Supplementary Materials, Figure S5)), enantiomeric purity over 99% (Supplementary Materials, Figure S6), and molar activity of 3.3–4.8 GBq/µmol. The activity yield (isolated, not decay-corrected) was 5.2 ± 0.5% (n = 3), with a synthesis time of ca. 70 min. The residual copper content, measured via ICP/MS, amounted to 0.7 ± 0.2 ppm and was below any level of concern according to the ICH Guideline of Elemental Impurities (Q3D) [46]. The concentration of methanol in the final preparation was analyzed via the standard GC procedure, and it was always below the recommended level of 3000 ppm [47]. The residual amount of DMAPOTf in the final preparation was determined via the previously developed method of capillary electrophoresis [48]; it was below 20 ppm. For this compound, there was no established limit available.

## 3. Experimental Section

### 3.1. Materials and Methods

Unless otherwise stated, reagents and solvents were commercially available and used without further purification. *N*,*N*-dimethylformamide (DMF), *N*,*N*-dimethylacetamide (DMA), 1,3-dimethyl-2-imidazolidinone (DMI), propylene carbonate (PC), *N*-Methyl-2-pyrrolidone (NMP), dimethyl sulfoxide (DMSO), methanol, 2-propanol, 1-butanol, acetone, acetonitrile, Cu(OTf)_2_(Py)_4_, 4-dimethylaminopyridine (DMAP), and trifluoromethanesulfonic acid (TfOH) were obtained from Sigma-Aldrich GmbH (Steinheim, Germany). Model arylBPin substrates, indole-4-boronic acid pinacol ester (**1**), 4-biphenylboronic acid pinacol ester (**2**), 2-methoxyphenylboronic acid pinacol ester (**3**), 3,4-dimethoxyphenylboronic acid pinacol ester (**4**), 4-methoxyphenylboronic acid pinacol ester (**5**), 4-hydroxyphenylboronic acid pinacol ester (**6**), and 2-aminophenylboronic acid pinacol ester (**7**) were obtained from Sigma-Aldrich GmbH (Steinheim, Germany). The precursor, tert-butyl-(*S*)2-((di-tert-butoxycarbonyl)amino)-3-(2-4,4,5,5-tetra-methyl-1,3,2-dioxaborolan-2-yl-4,5-dimethoxymethylphenyl)propanoate (**8**) for the synthesis of 6-L-[^18^F]FDOPA, was provided by WuXi AppTec Co (China). An authentic reference standard 6-D,L-FDOPA was obtained from ABX GmbH (Radeberg, Germany). The salt 4-(dimethylamino)pyridinium trifluoromethanesulfonate (DMAPOTf) was synthesized according to the previously reported procedure described by Antuganov et al. [29] using DMAP and TfOH. Deionized water (18.2 MOhm*cm) from an in-house Millipore Simplicity purification system (Merck KGaA, Darmstadt, Germany) was used for the preparation of all aqueous solutions. [^18^O]H_2_O (97% enrichment) was purchased from Global Scientific Technologies, Sosnovyi Bor, Russia. Oasis Wax 1cc cartridge (30 mg) and Sep-Pak Accell C18 Plus Light Cartridge (130 mg) were acquired from Waters Corporation (Millford, CT, USA). Oasis Wax 1 cc was conditioned with 10 mL of 0.5 M NaHCO_3_ followed by 10 mL of water. The Sep-Pak Accell C18 Plus Light Cartridge was conditioned with 5 mL of ethanol followed by 10 mL of water.

Radio-TLC analyses were carried out on precoated silica gel 60 F254 plates (Sorbfil, Lenchrom Ltd., St. Petersburg, Russia); the activity distribution was mapped using a Scan-RAM radioTLC scanner controlled using the chromatography software package Laura for PET (LabLogic, Sheffield, UK). The radiochemical conversion (RCC) measured via radioTLC was defined as the ratio of the product peak area to total peak area on the TLC. The RCC values obtained were not corrected for radioactive decay. To follow the course of radiofluorination, an aliquot of the crude reaction mixture was spotted onto a TLC plate, and the plate was eluted with CH_2_Cl_2_ (TLC system 1). The R_f_ values for [^18^F]fluoride, [^18^F]**1a**, and [^18^F]**8a** were 0.05, 0.51, and 0.49, respectively. For TLC analysis of the radiochemical purity of 6-L-[^18^F]FDOPA, the plate was eluted with methanol/acetic acid/HCl (9/0.5/0.5) (TLC system 2). The R_f_ values for [^18^F]fluoride and 6-L-[^18^F]FDOPA were 0.05 and 0.68, respectively.

The analytical HPLC system used, a Dionex ISC-5000, consisted of a gradient pump, a Rheodyne-type injector with a 20 μL loop, a variable-wavelength UV absorbance detector (set to 254 nm), and a 105-S model radiodetector (Carrol and Ramsey Associates, Berkeley, CA, USA). The UV and radioactivity detectors were connected in series, resulting in a delay of 0.1 min. The identity and radiochemical purity of [^18^F]FDOPA were determined using the following HPLC conditions. For system 1, the conditions were as follows: X-Bridge C18 HPLC column, 150 × 4.6 mm (Waters). For the eluent, the conditions were as follows: a mixture of a water solution of trifluoroacetic acid (0.1%) with acetonitrile using gradient elution conditions, a flow rate 2.0 mL/min. For the gradient, the conditions were as follows: 0–2.0 min 2% CH_3_CN isocratic; 2.0–13.0 min 2–95% CH_3_CN linear increase; 13.0–13.5 min 95–2% CH_3_CN linear decrease; 13.5–20.0 min 2% CH_3_CN isocratic (column equilibration). The R_t_ values for the reference standard 6-D,L-[^19^F]FDOPA, 6-L-[^18^F]FDOPA, and precursor **8** were 4.3, 4.4, and 10.4 min, respectively. The same HPLC conditions were used for monitoring the course of the radiofluorination reaction; the R_t_ value of the [^18^F]fluorinated intermediate [^18^F]**8a** was 12.8 min. The enantiomeric purity of the 6-L-[^18^F]FDOPA was evaluated using a Chirobiotic T (Astec) column eluted at 1.0 mL/min with a water solution of triethylaminoacetate (1%)/ethanol (90/10) (HPLC System 2). The R_t_ values for the L- and D-isomers of the reference standard 6-D,L-[^19^F]FDOPA were 4.3 and 5.0 min, respectively. The R_t_ value for the 6-L-[^18^F]FDOPA was 4.5 min. The content of copper in the final formulation was determined using a Varian 725-ES ICP Optical Emission Spectrometer. The residual amount of DMAPOTf was determined using the previously developed method of capillary electrophoresis (CE) [48]. CE analysis was performed using the Capel-105M system equipped with a UV spectrophotometric detector. The sample was loaded via pressure injection (10 s, 30 mbar) under the following conditions: column, 60 cm × 75 µm; background electrolyte of benzimidazole, 20 mmol/L and acetic acid, 40 mmol/L; UV, 230 nm; separation voltage, 25 kV, 30 °C. A stock solution of 4-dimethylaminopyridinium cations in normal saline at a concentration of 20 ppm was correspondingly prepared. The calibration standard was then prepared by means of serial dilution of the stock solution. Before analysis, the samples were diluted fourfold with deionized water. Under these conditions, the retention time (R_t_) for the 4-dimethylaminopyridinium cation was 3.5 min.

6-l-[^18^F]FDOPA was isolated using the HPLC package available on the GE Tracerlab FX C Pro module (General Electric Company, Cincinnati, OH, USA), consisting of a SYCAM S1122 pump, KNF UV detector, LAB LABOPORT, 2 mL injection loop, and β-radioactivity flow detector. The column used was an Ascentis RP-AMIDE, 250 × 10 mm, 5 µm (Supelco, Bellefonte, PA, USA) equipped with a guard column (an AJO-8327-S Security Guard Cartridge in a KJO-4282 Guard Cartridge Holder, Phenomenex, Torrance, CA, USA). The eluent used was water (0–6 min) that was subsequently replaced with 0.1% AcOH under a flow rate of 4 mL/min, and the R_t_ value for the 6-l-[^18^F]FDOPA was ~14 min.

### 3.2. Production of [^18^F]Fluoride

[^18^F]Fluoride was produced via the ^18^O(p,n)^18^F nuclear reaction via the irradiation of [^18^O]H_2_O (97% enrichment, Global Scientific Technologies, Petersburg, Russia) in a silver target (1.4 mL) with 16.4 MeV protons at a PETtrace 4 cyclotron (GE Healthcare, Uppsala, Sweden). The irradiated [^18^O]H_2_O was transferred from the target using a flow of helium, collected into a receiving vial of the automated synthesis module, and then loaded onto a modified (Figure 1) Oasis WAX 1 cc (30 mg) cartridge from the male side of the cartridge.

### 3.3. Radiolabeling of Model arylBPin Substrates 1–7

Model radiosyntheses of [^18^F]**1a**—of [^18^F]**7a** (Figure 2) were performed on the TRACERlab FX_FE_ Pro (GE Healthcare, Waukesha, WI, USA) that was modified for the purpose of this study, as shown in Figure 2. The process sequence is presented in Table 5. An aliquot of the irradiated ^18^O-water (150–250 MBq of [^18^F]F^−^ in 2 mL volume) was placed in the vial. Subsequently, the content of the vial was loaded onto the modified (Figure 1) Oasis WAX cartridge from the male side. This was followed by rinsing with different solvents (1.5 mL) and then drying with N_2_ gas for 2 min from the same side. The [^18^F]F^−^ was slowly eluted from the female side of the cartridge with a solution of DMAPOTf (25 μmol) in different eluting solvents (0.6 mL). All these operations (“back-flushing protocol”) were performed in the automated mode under low gas pressure. The eluate was collected in the reaction vessel (15 mL volume) prefilled with the solution of a certain amount of precursor (5–20 μmol) and Cu(OTf)_2_(Py)_4_ (5–30 μmol) in 0.6 mL of the reaction solvent. Radiofluorination was carried out for 20 min at 110 °C. The reaction was quenched by the addition of 80% aqueous EtOH to the cooled reaction mixture, and the RCC of the radiofluorination was determined by means of radioTLC.

### 3.4. Synthesis of 6-L-[^18^F]FDOPA under Remote-Controlled Operation

A solution of [^18^F]F^−^ (9.0–11.0 GBq) in [^18^O]H_2_O (1.4 mL) was loaded onto the modified (Figure 1) Oasis WAX 11 cc cartridge from the male side, followed by flushing with MeOH (1.5 mL) and drying with N_2_ gas for 2 min. [^18^F]F^−^ was eluted from the female side of the cartridge with a solution of DMAPOTf (25 μmol) in DMI/DMA 1/1 (0.6 mL) into the reaction vessel (5 mL volume) prefilled with a solution of 20 µmol of precursor **8** and 20 µmol of Cu(OTf)_2_(Py)_4_ in 2-PrOH/DMI 1/1 (0.6 mL). The reaction mixture was heated at 65 °C for 10 min and at 110 °C for 10 min with stirring. The reaction vessel was cooled down to 40 °C. An amount of 9 mL H_2_O was added to the reaction mixture, and the resulting solution was loaded onto a Sep-Pak C18 Plus cartridge (130 mg). The cartridge was then washed with 5 mL of H_2_O, and radiolabeled intermediate [^18^F]**8a** was eluted with MeOH (1.5 mL). The mixture of 6 N HCl (0.4 mL) and 0.25 M ascorbic acid (0.2 mL) was added, and hydrolysis was carried out for 5 min at 110 °C under stirring by nitrogen flow with an open waste and 5 min with a closed waste. The aliquot of the reaction mixture was analyzed by means of radioHPLC (HPLC system 1).

### 3.5. Automated Synthesis of 6-L-[^18^F]FDOPA

Radiosynthesis was performed on the TRACERlab FX N Pro (GE Healthcare, Waukesha, WI, USA) using the same amounts of the reagents that were placed in the reagent vials of the module. The radionuclide (10–11 GBq) was transferred from the target in a water bolus by means of helium flow and collected into the receiving vessel. The content of the vessel was pushed through the modified (Figure 1) Oasis Wax 1cc cartridge from the male side, and the cartridge was rinsed with 2 mL of MeOH from the same side, followed by drying within 3 min. The trapped [^18^F]fluoride was then eluted from the female side with a solution of 6.5 mg of DMAPOTf ((25 μmol) in 0.6 mL of DMI/DMA 1/1; the eluate was collected in the reaction vessel prefilled with a solution of 14 mg (20 μmol) of **8** and 14 mg of Cu(OTf)_2_(Py)_4_ (20 μmol) in 0.6 mL of 2-PrOH/DMI 1/1. The reaction mixture was heated at 65 °C for 10 min, followed by a second round of heating at 110 °C for 10 min under stirring. The reaction vessel was then cooled down to 40 °C and diluted with 9 mL of H_2_O with stirring. The resulting solution was pushed through the Sep-Pak Accell C18 Plus Light Cartridge (130 mg) followed by 5 mL of water. The protected 6-L-[^18^F]FDOPA ([^18^F]**8a**) was eluted with 1.5 mL of MeOH into the second reactor; this was followed by 3 min of heating (110 °C) under nitrogen flow for partial evaporation of the MeOH up to a volume of 0.5 mL. To the obtained solution, 0.4 mL of 6 N HCl and 0.2 mL of 0.25 M ascorbic acid were added, and hydrolysis was carried out for 3 min at 110 °C with stirring, following heating at the same temperature under nitrogen flow to reduce the volume of the reaction mixture to 0.5 mL. The vessel was then cooled down to 40 °C, and the crude reaction mixture was diluted with 1.5 mL of the HPLC eluent, 0.1% CH_3_COOH.

### 3.6. Semipreparative Purification of 6-L-[^18^F]FDOPA

The diluted hydrolysate (total volume, c.a. 1.8–1.9 mL) was loaded onto a 2 mL HPLC loop and injected into an Ascentis RP-AMIDE semipreparative HPLC column (5 µm, 250 × 10 mm (Supelco)). The column was equipped with a guard column to remove residual copper (the AJO-8327-S Security Guard Cartridge in the KJO-4282 Guard Cartridge Holder (Phenomenex)). The column outlet was connected to a UV absorbance detector (λ = 254 nm) in series with a radioactivity detector (TRACERlab FX C Pro module (GE Healthcare). After running the HPLC with pure water (0–6 min), the mobile phase was changed to 0.1% CH_3_COOH at a flow rate of 4 mL/min, producing a radioactive fraction (4–5 mL volume) corresponding to that of pure 6-L-[^18^F]FDOPA (R_t_ from 14 to 15 min). The product fraction with pH 4 was collected into a vented sterile vial prefilled with 5 mL of sterile normal saline through the 0.22 μm Millipore filter.

## 4. Conclusions

In this study, we demonstrated the utility of DMAPOTf, an efficient PTC for the copper-mediated radiofluorination of arylBPin substrates in an automated mode. The translation of our previous results obtained in a remote-controlled apparatus with manual intervention [30] to the TRACERlab automation platform was not straightforward. The main difficulty was the implementation of the “back-flushing protocol” into the automated operation with the rarely used Oasis WAX 1 cc barrel-type cartridge that was selected in our previous work [30]. The problem was solved via a slight modification of the cartridge design involving the insertion of a replaceable PEEK liner. After the adjustment of the reagent amounts and solvents, the novel protocol was successfully applied in the radiofluorination of a series of model substrates in automated mode on a TRACERlab FX_FE_ Pro adapted for nucleophilic radiofluorination. The practical applicability of the methodology was demonstrated in an automated synthesis of 6-L-[^18^F]FDOPA; a radiotracer was obtained in an AY (isolated, not decay-corrected) of 5.2 ± 0.5% (n = 3) with a synthesis time of ca. 70 min on the TRACERlab FX N Pro automation platform. The obtained AY was comparable with one reported by Scott’s group (6 ± 1%) [43] using the same labeling precursor 8, while a slightly higher AY of 6-L-[^18^F]FDOPA (14.5 ± 0.5%) was achieved in our previous work [24] using commercially available Bu_4_NOTf as the PTC. In conclusion, the operationally simple and practical azeotropic drying-free method, using DMPAOTF elution, that we developed in this work proved to be useful for the automated production of PET tracers, enhancing the practical utility of Cu-mediated radiofluorinations.

## Figures and Tables

**Table 1:** The effect of rinsing solvent on the EE and RCC in the radiofluorination of **1**; conditions: elution of [^18^F]F^−^ from WAX 1cc by DMAPOTf (25 μmol) in 0.6 mL of DMA; fluorination: 10 μmol of **1**, 10 μmol of Cu(OTf)_2_Py_4_ in 0.6 mL of DMA, 110 °C, 20 min; no air atmosphere.

Entry	Rinsing Solvent	EE ± SD, % (n = 3)	RCC ± SD, % (n = 3)
1	2-Propanol	73 ± 1	13 ± 1
2	Acetone	68 ± 1	43 ± 2
3	Acetonitrile	75 ± 6	36 ± 13
4	*n*-Butanol	78 ± 2	47 ± 4
5	Methanol	76 ± 8	63 ± 5

**Table 2:** The effect of precursor/catalyst ratio on the RCC in fluorination of **1**; MeOH as a rinsing solvent; WAX 1cc eluent: DMAPOTf (25 μmol) in 0.6 mL of DMA; fluorination: 5–20 μmol of 1 and 5–30 μmol of Cu(OTf)_2_Py_4_ in 0.6 mL of DMA, 110 °C, 20 min; no air atmosphere.

Entry	Precursor 1, μmol	Catalyst, μmol	RCC ± SD, % (n = 3)
1	5	5	26 ± 6
2	10	10	63 ± 5
3	10	20	61 ± 4
4	20	10	45 ± 9
5	20	20	75 ± 3
6	20	30	58 ± 1

**Table 3:** Effect of different eluting and reaction solvents on the EE and RCC in the automated mode; conditions: WAX 1 cc eluent: 25 μmol of 4-DMAPOTf in 0.6 mL of the solvent (raw 2); 20 μmol of **1** and 20 μmol of Cu(OTf)_2_Py_4_ in 0.6 mL of the solvent (row 3) *; 110 °C; 20 min.

Entry	Eluting Solvent	Reaction Solvent *	EE ± SD, % (n = 3)	RCC ± SD, % (n = 3)
1	DMA	DMA	78 ± 5	75 ± 3
2	DMF	DMF	85 ± 1	7 ± 1
3	PC	PC	29 ± 3	33 ± 2
4	NMP	NMP	68 ± 2	53 ± 8
5	DMI	DMI	58 ± 1	92 ± 1
6	DMSO	DMSO	68 ± 2	0
7	DMA	DMI	78 ± 5	81 ± 2
8	DMA/DMI 1/1	DMI	75 ± 4	89 ± 2

* The solvent to solubilize **1**, and Cu catalyst in the reaction vessel.

**Table 4:** The results of Cu-mediated radiofluorination of **8**: [^18^F]fluoride elution from WAX by solution of DMAPOTf(25 μmol) in 0.6 mL of DMI/DMA 1/1; fluorination: 10–20 μmol of **8**, 20 μmol of Cu(OTf)_2_Py_4_ in 0.6 mL of DMI or DMI/2-PrOH 1/1; no access to atmospheric air; the number experiments was limited due to the high cost of the labeling precursor.

Entry	Precursor, Μmol	Fluorination Solvent *	Reaction T, °C/ Time, min	RCC [%] RadioTLC (n = 1)	Module
1	10	DMI	110/20	7	FX FE
2	20	DMI	110/20	18	FX FE
3	20	DMI/2-PrOH 1/1	110/20	32	FX FE
4	20	DMI/2-PrOH 1/1	110/20	38	FX FE
5	20	DMI/2-PrOH 1/1	65/10 110/10	77	Remote-controlled
6	18	DMI/2-PrOH 1/1	65/10 110/10	70	Remote-controlled
7 **	18	DMI/2-PrOH 1/1	65/10 110/10	65	Remote-controlled

* The solvent to solubilize **8** and Cu catalyst in the reaction vessel; ** 22 μmol of DMAPOTf in 0.6 mL of DMI/DMA for elution and 18 μmol of Cu(OTf)_2_Py_4._

**Table 5:** Process sequence for the automated radiofluorination of model arylBPin substrates **1–7** using the modified GE TRACERlab FX_FE_-Pro module (Figure 2).

Entry	Process	Activated Path/Function
1	Loading of [^18^F]fluoride onto the Wax1cc cartridge	V21-V30-Wax1cc-V31-waste bottle
2	Washing of Wax1cc cartridge with MeOH (2 mL)	V16-V21-V30-Wax1cc-V31-waste bottle
3	Elution of [^18^F]fluoride from the cartridge into the RV	V16-V1-V31-Wax1cc-V30-reactor
4	Radiofluorination, RV, 110 °C, 20 min stirring	-
5	Addition of 80% EtOH (2ml)	V16-V2-RV
6	Sampling	V16-V2-RV

## Data Availability

The data presented in this study are available on request from the corresponding author.

## Supplementary Materials

The following supporting information can be downloaded at: https://www.mdpi.com/article/10.3390/molecules29143342/s1, Figure S1: Reaction temperature (A) and time (B) screens; Figure S2: RadioTLC data for the radiofluorination of 1; Figure S3: RadioTLC data for the radiofluorination of 8 in the remote-controlled synthesis of 6-L-[^18^F]FDOPA; Figure S4: RadioTLC data for the radiofluorination of 8 in the automated synthesis; Figure S6: Radio HPLC analysis of the enantiomeric purity of 6-L-[^18^F]FDOPA; Figure S7: Purification of 6-L-[^18^F]FDOPA by preparative HPLC.
