# Supplementary material for: Automation of Copper-Mediated 18F-Fluorination of Aryl Pinacol Boronates Using 4-Dimethylaminopyridinium Triflate
Source: Molecules. 2024 Jul 16;29(14):3342. doi: 10.3390/molecules29143342 (PMC11279627; doi:10.3390/molecules29143342)
Supplement: Supplementary file 1 [file molecules-29-03342-s001.zip › molecules-3062599-supplementary.pdf]

Supporting information for:

Automation of copper-mediated ^{18}F-fluorination of aryl pinacol boronates using 4-dimethylaminopyridinium triflate

M. A. Nadporojskii[1], V. V. Orlovskaya[2], O. S. Fedorova[2], D. S. Sysoev[1], R. N. Krasikova[2#]

[1]Granov Russian Research Center of Radiology and Surgical Technologies, 197758 St.-Petersburg, Russia;

[2]N.P.Bechtereva Institute of the Human Brain, 197376 St.-Petersburg, Russia;

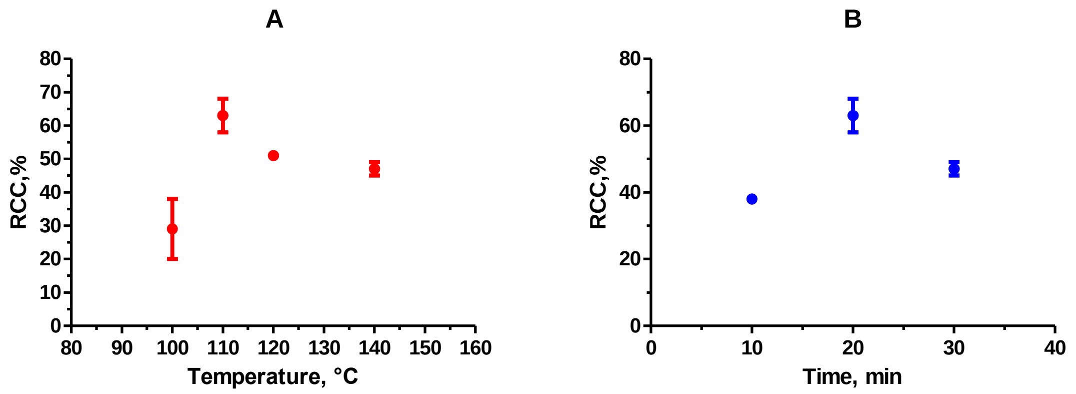

Figure S1. Reaction temperature (A) and time (B) screens. Conditions: OASIS WAX 1 cc eluent - 25 µmol of DMAPTfO$^-$ in 0.6 mL of DMA; 10 µmol of precursor **1** and 10 µmol of $Cu(OTf)_2Py_4$ in 0.6 mL of DMA; 100-140 °C ; 10-30 min)

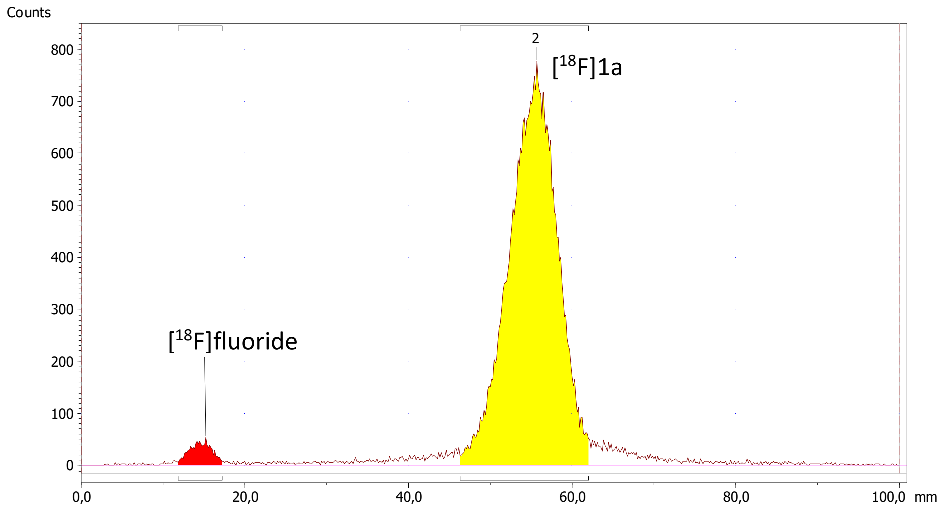

Figure S2. RadioTLC data for the radiofluorination of 1 (remote-controlled synthesis); eluent CH_2Cl_2, TLC system 1. RCC 96%.

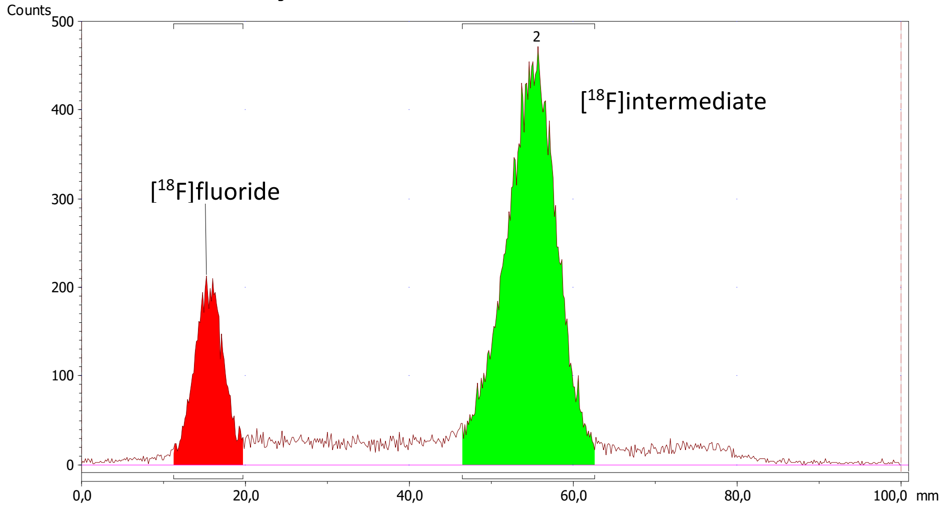

Figure S3. RadioTLC data for the radiofluorination of 8 in the remote-controlled synthesis of 6-L-[^{18}F]FDOPA; eluent CH_2Cl_2, TLC system 1. RCC 77%.

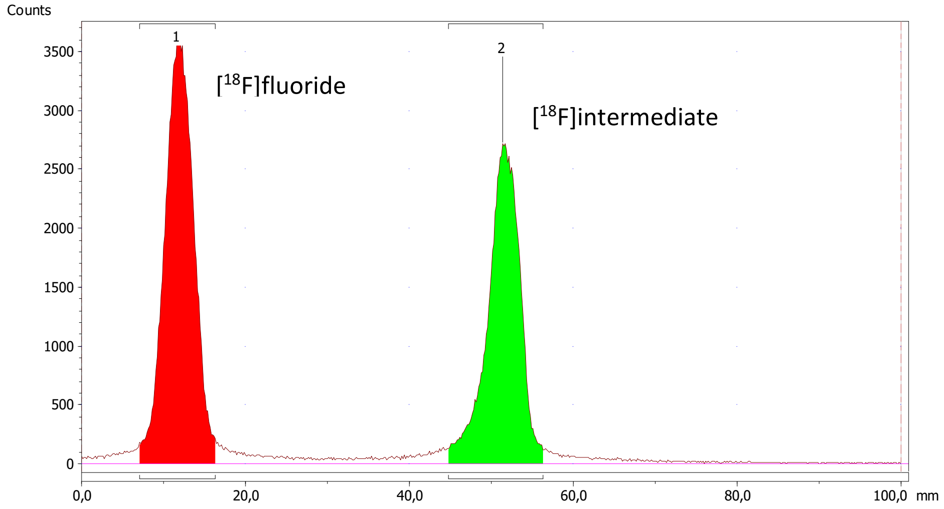

Figure S4. RadioTLC data for the radiofluorination of **8** in the automated synthesis of 6-L-[^{18}F]FDOPA using TRACERlab FX N Pro; eluent CH_2Cl_2, TLC system 1. RCC 45%.

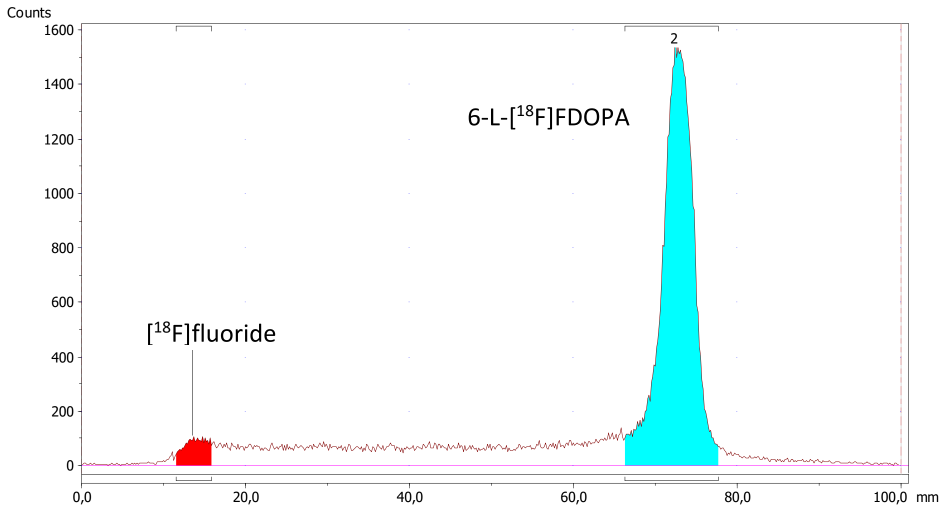

Figure S5. RadioTLC anaylysis of radiochemical purity of formulated 6-L-[^{18}F]FDOPA; eluent: methanol/acetic acid/HCl (9/0.5/0.5), TLC system 2. RCP>99%.

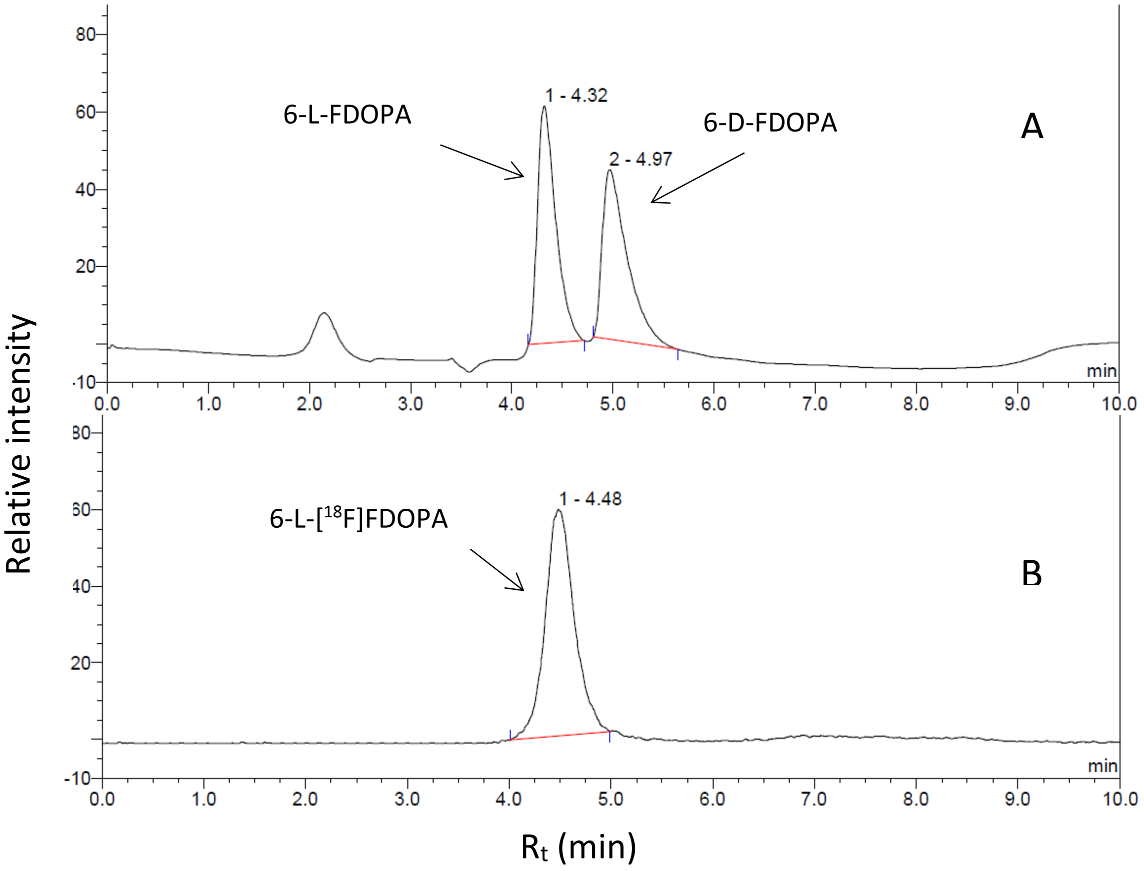

Figure S6. Radio HPLC analysis of the enantiomeric purity of 6-L-[^{18}F]FDOPA (HPLC system 2, UV 254 nm). A) UV chromatogram of authentic reference 6-D,L -DOPA; B) radioHPLC chromatogram of the formulated 6-L-[^{18}F]FDOPA.

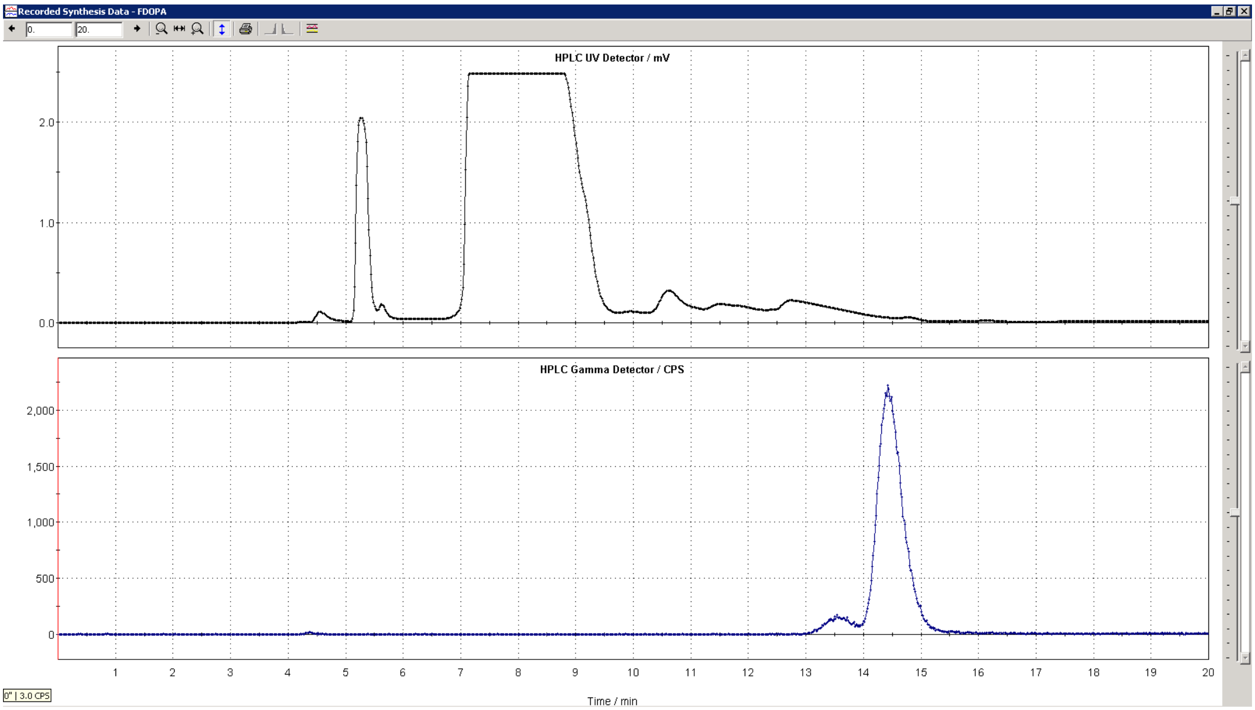

Figure S7. Purification of 6-L-[^{18}F]FDOPA by preparative HPLC on the HPLC column Ascentis RP-AMIDE, 5 µm, 250×10 mm (Supelco), flow rate 4 ml/min; top: UV trace, λ=254 nm; bottom: radioactivity trace.
